# Intranasal Administration of Nanovectorized Docosahexaenoic Acid (DHA) Improves Cognitive Function in Two Complementary Mouse Models of Alzheimer’s Disease

**DOI:** 10.3390/antiox11050838

**Published:** 2022-04-25

**Authors:** Charleine Zussy, Rijo John, Théo Urgin, Léa Otaegui, Claire Vigor, Niyazi Acar, Geoffrey Canet, Mathieu Vitalis, Françoise Morin, Emmanuel Planel, Camille Oger, Thierry Durand, Shinde L. Rajshree, Laurent Givalois, Padma V. Devarajan, Catherine Desrumaux

**Affiliations:** 1 MMDN, University Montpellier, EPHE, INSERM, 34095 Montpellier, France; charleine.zussy@umontpellier.fr (C.Z.); theo.urgin@etu.umontpellier.fr (T.U.); lea.otaegui@etu.umontpellier.fr (L.O.); geoffrey.canet.1@ulaval.ca (G.C.); mathieu.vitalis@umontpellier.fr (M.V.); laurent.givalois@umontpellier.fr (L.G.); 2 Department of Pharmaceutical Sciences and Technology, Institute of Chemical Technology, Deemed University, Mumbai 400019, India; php14rj.john@pg.ictmumbai.edu.in (R.J.); rshinde1@abiteccorp.com (S.L.R.); pv.devarajan@ictmumbai.edu.in (P.V.D.); 3 IBMM, Pôle Chimie Balard Recherche, Université de Montpellier, CNRS, ENSCM, 34095 Montpellier, France; claire.vigor@umontpellier.fr (C.V.); camille.oger@umontpellier.fr (C.O.); thierry.durand@umontpellier.fr (T.D.); 4 Centre des Sciences du Goût et de l’Alimentation, AgroSup Dijon, CNRS, INRAE, Université de Bourgogne Franche-Comté, 21000 Dijon, France; niyazi.acar@inrae.fr; 5 Department of Psychiatry and Neurosciences, Faculty of Medicine, Laval University, CR-CHUQ, Québec City, QC G1V 0A6, Canada; francoise.morin@crchudequebec.ulaval.ca (F.M.); emmanuel.planel@fmed.ulaval.ca (E.P.); 6 LIPSTIC LabEx, 21000 Dijon, France

**Keywords:** omega-3 fatty acids, docosahexaenoic acid, curcumin, Alzheimer’s disease, amyloid-β peptide, intranasal, microemulsion, cognitive impairment, Tau protein

## Abstract

Polyunsaturated fatty acids (PUFAs) are a class of fatty acids that are closely associated with the development and function of the brain. The most abundant PUFA is docosahexaenoic acid (DHA, 22:6 *n*-3). In humans, low plasmatic concentrations of DHA have been associated with impaired cognitive function, low hippocampal volumes, and increased amyloid deposition in the brain. Several studies have reported reduced brain DHA concentrations in Alzheimer’s disease (AD) patients’ brains. Although a number of epidemiological studies suggest that dietary DHA consumption may protect the elderly from developing cognitive impairment or dementia including AD, several review articles report an inconclusive association between omega-3 PUFAs intake and cognitive decline. The source of these inconsistencies might be because DHA is highly oxidizable and its accessibility to the brain is limited by the blood–brain barrier. Thus, there is a pressing need for new strategies to improve DHA brain supply. In the present study, we show for the first time that the intranasal administration of nanovectorized DHA reduces Tau phosphorylation and restores cognitive functions in two complementary murine models of AD. These results pave the way for the development of a new approach to target the brain with DHA for the prevention or treatment of this devastating disease.

## 1. Introduction

Cognitive impairment due to Alzheimer’s disease (AD) has emerged over the past 20 years as a major factor, reducing the quality of life of patients and their caregivers, and is a huge threat to healthcare resources. Sporadic forms of AD are thought to result from the interaction of aging, genetic factors—especially genes involved in lipid metabolism and transport—and environmental factors [1]. They are expected to increase dramatically in the next decades. Among the modifiable environmental factors modulating the risk for cognitive impairment and dementia, there is an increasing interest in the impact of dietary nutrients [2].

Polyunsaturated fatty acids (PUFAs) are a class of fatty acids closely associated with the development and function of the brain. The most abundant PUFA is docosahexaenoic acid (DHA, 22:6 *n*-3). DHA is a major constituent of cell membranes in the brain and plays a major role in ensuring optimal neuronal functions [3]. It regulates membrane fluidity and controls the function of synaptic membrane-associated proteins. DHA can be provided to the body from dietary intakes or from the bioconversion of the essential α-linolenic acid (ALA, 18:3 *n*-3) in the liver, which is marginal in humans [4]. Low plasma concentrations of DHA have been associated with impaired cognitive function, low hippocampal volumes, and increased amyloid deposition in the brain. In humans, several studies have reported reduced brain DHA concentrations in AD patients [5].

Since Western diets contain low amounts of DHA, supplementation appears as a promising strategy for the primary prevention of AD. A number of studies have shown that long-term DHA supplementation has anti-amyloidogenic properties in AD animal models, thereby diminishing neuronal loss and increasing cognitive functions [6,7,8,9]. In humans however, although a number of epidemiological studies suggest that dietary DHA consumption may protect the elderly from developing cognitive impairment or dementia including AD, several review articles report an inconclusive association between omega-3 PUFA intake and cognitive decline [2,4,10]. One possible explanation is that dietary DHA occurs most commonly in the form of triglycerides (fish oil, algal oil) or phospholipids (krill oil), which are not predominantly absorbed by the brain.

Current supplements are also available as ethyl ester (Lovaza, Vascepa) and free fatty acid (Epanova) forms. However, none of these preparations significantly enrich brain DHA in the recommended doses, although they enrich most other tissues. This is apparently because these supplements are absorbed predominantly as triglycerides, whereas the preferred carrier of DHA across the blood–brain barrier (BBB) is the lysophosphatidylcholine form [11,12]. Although krill oil has about 40% phospholipids by weight, the DHA in the phospholipids are present in the SN2 position and are released as free fatty acids during digestion by pancreatic phospholipase A_2_. The released DHA is subsequently absorbed as triglycerides, and therefore will not significantly enrich the brain. Also, because DHA has six double bonds and five methylene carbons, another point that needs to be considered is its high susceptibility to oxidation. DHA is more readily oxidized than linoleic, linolenic, and arachidonic acids [13], thus its oxidation state needs to be considered and carefully controlled to avoid discrepancies in preclinical and clinical studies.

To summarize, because DHA is highly oxidizable and its accessibility to the brain is limited by the BBB, there is a pressing need for new strategies to improve DHA brain supply.

Direct intranasal administration has recently emerged as a beneficial alternative to the oral and parenteral routes for central nervous system (CNS) targeting. This route of administration bypasses the BBB and allows the passage of molecules along the olfactory and trigeminal nerves, which are in direct contact with the CNS [14,15]. The efficacy of this system for CNS targeting has recently been validated in clinical trials for cerebral insulin delivery [16] and is being evaluated for other drugs such as acetylcholinesterase inhibitors [17]. The main advantages of the intranasal route of administration are (i) its accessibility, (ii) non-invasiveness, and (iii) its very low metabolic activity compared to the gastrointestinal wall, leading to high bioavailability [14,15].

In the present study, we addressed for the first time the impact of an intranasal administration of DHA-enriched microemulsions on oxidative stress, neuroinflammation, Tau phosphorylation, and cognitive functions in two complementary murine models of AD. The impact of DHA association with the polyphenolic compound Curcumin (CUR) as an antioxidant in microemulsions was also addressed [18,19].

## 2. Materials and Methods

### 2.1. Materials

Curcumin, DHA rich oil (INCROMEGA DHA 500 TG SR), and Capmul MCM (a mono- and diglyceride emulsifier) were obtained free of charge from Konark herbals and Healthcare (Mumbai, India, assay 99.9%), Croda Chemicals (Mumbai, India, Private Limited), and Abitec Corporation Ltd. (Mumbai, India), respectively.

The 15-A_2t_-isoprostane (15-A_2t_-IsoP) was purchased from Cayman Chemical (Ann Arbor, MI, USA). The 5(*S*)-5-F_3t_-IsoP and the 4(*RS*)-4-F_4t_-neuroprostane (4(*RS*)-4-F_4t_-NeuroP) were synthesized and fully characterized according to published procedures [20,21]. The 13(*RS*)-13-F_4t_-NeuroP was a gift from Pr Douglass F. Taber from the University of Delaware.

Tween 80, ethanol, and propylene glycol were obtained from Merck India Pvt. Ltd (Mumbai).

### 2.2. DHA-Loaded Microemulsions

The microemulsions (MEs) were prepared and characterized by Dr P.V. Devarajan’s team as previously described [18]. They comprised 60% Tween80:ethanol (3:1), 10% oil phase, and 30% water by weight. The oil concentration was maintained at 10% and comprised either DHA-rich oil:Capmul MCM (1:1)—referred to as DHA ME—or only Capmul MCM—referred to as Capmul ME. To evaluate the effect of Curcumin in microemulsions, 5 mg/mL were added in Capmul ME and DHA ME. These preparations were referred to as CUR ME and CURDHA ME, respectively.

The DHA concentration in MEs was determined according to previously described procedures [22,23]. Lipids were extracted according to the Folch method [24]. The total lipids were extracted with 5 mL of a chloroform/methanol mixture (2:1, *v*/*v*). The samples were then centrifuged at 3000 rpm for 3 min and the lower organic phase was isolated and evaporated to dryness under a stream of nitrogen. Finally, the total lipids were dissolved in hexane and stored under nitrogen at −20 °C until further analyses. The fatty acids were transmethylated using boron trifluoride in methanol according to Morrison and Smith [25]. The fatty acid methyl esters formed by transmethylation were extracted with hexane. They were analyzed on a Trace 1310 gas chromatograph (Thermofisher, Waltham, MA, USA) equipped with a CPSIL-88 column (100 m × 0.25 mm i.d., film thickness 0.20 µm; Varian, Les Ulis, France). Hydrogen was used as a carrier gas (inlet pressure 210 kPa). The oven temperature was held at 60 °C for 5 min, increased to 165 °C at 15 °C/min and held for 1 min, then increased to 225 °C at 2 °C/min, and finally held at 225 °C for 7 min. The injector and the flame ionization detector were maintained at 250 °C. DHA methyl esters were identified by comparison with commercial and synthetic standards. The data were computed using the EZChrom software (Agilent Technologies, Massy, France).

### 2.3. Assessment of the Antioxidant Capacity of Microemulsions

The oxygen radical absorbance capacity (ORAC) assay was performed to assess the antioxidant capacity of MEs, using Trolox as a positive control [26]. Briefly, 25 μL of MEs or Trolox were pipetted in a 96-well white microplate (with clear bottom), whereas phosphate buffer (25 μL, 75 mM, pH 7.4) was transferred in the negative control wells. Fluorescein solution (150 μL, 25 nM) was added in each well and the plate was incubated in the dark at 37 °C for 30 min. Subsequently, AAPH solution (25 μL, 0.15 M) was added in the positive control and the antioxidant wells, whereas phosphate buffer (25 μL) was pipetted in the negative control, and the fluorescence was measured every 2 min over a period of 2 h (485/20 nm excitation, 525/20 nm emission) using a Fluoroskan Ascent microplate reader (ThermoScientific, Waltham, MA, USA). The non-enzymatic DHA oxidation that occurred in the microemulsions was measured as described below (Section 2.4.4).

### 2.4. In Vivo Evaluation of Microemulsions

#### 2.4.1. Animals

All mice were housed in a standard animal facility (12L/12D cycle, 21 ± 2 °C) with free access to water and food (standard chow diet, SAFE Diets, Augy, France). All procedures were conducted in strict adherence to the European Union Directive of 22 September 2010 (2010/63/UE). This project followed the specific French national guidelines on animal experimentation and well-being; the National French Animal Welfare Committee and the local committee at the University of Montpellier approved all protocols (authorization: CEEA-LR-6914). All efforts were made to minimize the number of animals used, potential pain, suffering, and distress.

Two complementary AD models were used in the present study: the acute oAβ_25–35_ model and the J20 transgenic model.

#### 2.4.2. oAβ_25–35_ Model

Aβ_25–35_ and scrambled Aβ_25–35_ peptide (ScAβ_25–35_) (Bachem, Bubendorf, Switzertland) were dissolved in sterile water at a concentration of 3 μg/μL (soluble form) and stored at −20 °C. Aβ_25–35_ was aggregated by in vitro incubation at 37 °C for 4 days to obtain a solution of Aβ_25–35_ oligomers (oAβ_25–35_). Scrambled peptide, i.e., a control peptide that does not aggregate as Aβ_25–35_, was incubated at 37 °C for 4 days and was used for the sham treatment [27].

Eight-week-old C57Bl/6J males were purchased from Janvier Labs (Le Genest-Saint-Isle, France). A group received an intracerebroventricular (icv) injection of incubated ScAβ_25–35_ (9 μg/mouse) (Scr, control group) and another group received an icv injection of oAβ_25–35_ peptide (9 μg/mouse) (Aβ group). As previously described [28], the animals were anesthetized with isoflurane and injected directly into the lateral ventricles using a Hamilton syringe (Phymep, Paris, France) (coordinates: AP: −1 mm, L: ±1.5 mm, DV: −3.5 mm) [29]. Three days after icv injections (day 3), the control and Aβ groups were divided into subgroups to receive treatments, which consisted of one intraperitoneal (IP, 10 mL/kg) or intranasal (IN, 10 µL/mouse) administration of either Capmul ME (control groups: IP n = 14; IN, n = 17/Aβ groups: IP n = 14; IN, n = 15), CUR-ME (control groups: IP n = 8; IN, n = 7/Aβ groups: IP n = 11; IN, n = 8), DHA-ME (control groups: IP n = 8; IN, n = 9/Aβ groups: IP n = 9; IN, n = 9), or CURDHA-ME (control groups: IP n = 8; IN, n = 7/Aβ groups: IP n = 11; IN, n = 13) per day for 4 days. The administered dose was 2 mg/kg for CUR and 10 mg/kg for DHA. Vehicle mice received Capmul ME and served as negative controls.

Short-term spatial memory was tested the day after the last treatment using the Y-maze test, and the animals were then sacrificed by decapitation (day 7). The hippocampus and prefrontal cortex were rapidly collected for Western blot analysis and oxylipins quantification.

#### 2.4.3. J20 Transgenic Model

The J20 mouse model, which overexpressed human APP containing the Swedish (K670N/M671L) and Indiana (V717F) mutations under the control of platelet-derived growth factor-beta (PDGF-â) promoter [30], was used as a chronic AD model in the present study. Hemizygous J20 founders were purchased from the Jackson Laboratories. A Material Transfer Agreement (#UM140255-01) was signed with the Gladstone Institute for their breeding and use in the present study. All mice were on the C57BL/6J background and backcrossed > 10 generations. The genotyping has been described previously [28]. Three-month-old female J20 mice and WT littermates were used in this study. We deliberately chose to start treating the mice at a very early stage of the disease before the accumulation of extracellular Aβ, since we wanted to evaluate its potential in the context of disease prevention. Mice were treated 4 weeks, 3 times/week, with either CURDHA-ME (n = 13 J20 mice) or Capmul ME (n = 18 WT and n = 12 J20 mice), and behavioral phenotyping was performed at the end of the treatment period. The animals were then sacrificed by decapitation. The hippocampus and prefrontal cortex were rapidly collected for Western blot analysis and oxylipins quantification.

#### 2.4.4. Behavioral Tests

##### Y-Maze Test

Spontaneous alternation behavior, which is a measure of short-term spatial working memory, was tested using a Y-maze following a previously described protocol [28]. Each animal was video tracked (Noldus EthoVisonXT14 video-tracking system, Wageningen, The Netherlands) and the percentage of alternation was calculated as (actual alternations/maximum alternations) × 100. The apparatus was cleaned with diluted ethanol (50%) between each session.

##### Splash Test

In this test, the dorsal coat of mice is squirted with a 10% sucrose solution (*w*/*v*), which initiates grooming behavior. The total grooming duration was recorded for 5 min, as an index of self-care and motivational behavior, and considered any apathetic behavior as depressive symptoms [31,32].

##### O-Maze Test

The O-maze (OM) apparatus consists of two open (stressful) and two enclosed (protecting) elevated areas (40 cm above the floor) that form a circle (diameter of 46 cm). Each animal was video tracked (Noldus EthoVisonXT14 video-tracking system) for 5 min and the number of entries and time spent in each area was calculated. The time spent in enclosed versus open areas indicated anxiety level [33]. The apparatus was cleaned with diluted ethanol (50%) between each session.

#### 2.4.5. Western Blots

Mice were sacrificed by decapitation. Their hippocampus was micro-dissected, weighed, immediately frozen in liquid nitrogen, and stored at −80 °C. The tissues were sonicated with a VibraCell (Sonics & Materials, Newton, MA, USA) in a RIPA lysis buffer (50 mM tris, 1% NP-40, 150 mM NaCl, 1 mM EDTA) containing protease and phosphatase inhibitors (Roche Diagnostics GmbH, Mannheim, Germany) and centrifuged at 4 °C, as previously described [34]. The supernatants were collected, and the protein concentration was measured using a BCA kit (Thermofisher, Illkirch-Graffenstaden, France). Sixty micrograms from each sample were taken for Western blot analysis. The samples were boiled for 5 min, separated in SDS-polyacrylamide gel (12%), and transferred to a polyvinylidene difluoride membrane (Whatman, Versailles, France). The membrane was incubated overnight (4 °C) with the primary antibody, was rinsed, and then incubated for 2 h with the appropriate horseradish peroxidase-conjugated secondary antibody (Sigma–Aldrich, Saint Quentin Fallavier, France). Peroxidase activity was revealed by using enhanced chemiluminescence (ECL) reagents (Luminata Crescendo, Millipore), Molsheim, France. The intensity of peroxidase activity was quantified using Image-J software (NIH, Bethesda, USA). Beta-tubulin (β-tub) and beta-actin were used as loading controls for GFAP and dPP2Ac quantification, respectively, whereas the total protein was used for phosphorylated protein isoforms.

The following antibodies were used: mouse anti-GFAP (1/1000, Sigma Aldrich Saint Quentin Fallavier, France), mouse anti-phospho Tau (phospho-threonine 231, clone AT180, 1/1000, Thermofisher, Illkirch Graffenstaden, France), mouse anti-phospho Tau CP13 (phospho-serine 202, 1/1000 gift from Dr. Peter Davies, Albert Einstein University, New York, NY, USA), rabbit anti-Tau C (total Tau, 1/1000, DAKO), mouse anti-glycogen synthase kinase-3β (GSK-3β) (1/1000, BD Transduction Laboratories), rabbit anti-phospho-GSK-3β (phospho-serine 9, 1/1000, Thermofisher), rabbit anti-phospho-GSK-3β (phospho-tyrosine 216, 1/1000, Thermofisher), rabbit anti-stress-activated protein kinase (SAPK)/c-Jun N-terminal kinase (JNK) (1/1000, Thermofisher), rabbit anti-phospho-SAPK/JNK (phospho-threonine 183/phospho-tyrosine 185, 1/1000, Thermofisher), mouse anti-demethylated PP2AC (protein phosphatase 2A, subunit C) (1/1000, Santa Cruz, Dallas, USA), mouse anti-beta-actin (1/10,000, Sigma Aldrich), and mouse anti-beta-tubulin (β-tub, 1/5000, Sigma Aldrich).

#### 2.4.6. Oxylipins Quantification

Non-enzymatic oxygenated polyunsaturated fatty acid metabolites (NEO-PUFAs) were extracted from prefrontal cortex tissue or microemulsions as previously published [35]. The qualitative and quantitative profile of these non-enzymatic lipid mediators was determined in the mouse brain by micro-LC-MS/MS as previously described [36]. Their concentration was established by calibration curves calculated from the area ratio of analytes and the internal standards. Data processing was performed using a MultiQuant 3.0 software (Sciex Applied Biosystems, Framingham, MA, USA).

### 2.5. Statistical Analysis

The data are presented as mean ± SEM and analyzed using 1-way or 2-way analysis of variance followed by a Tukey’s multiple comparison test (GraphPad Prism 5.0, San Diego, CA, USA). A *p* value of <0.05 was considered significant. Before each analysis of variance, the Gaussian distribution was systematically evaluated and validated by a Kolmogorov–Smirnov test (GraphPad Prism 5.0).

## 3. Results

### 3.1. DHA Stability and Antioxidant Capacity of MEs

Given that DHA is highly susceptible to oxidation, we first evaluated its stability in MEs containing Curcumin or not. As shown in Table 1, the amount of DHA present in DHA ME decreased rapidly over storage at 4 °C under nitrogen: by 9% after 1 month, 45% after 2 months, and 80% after 3 months. In contrast, the amount of DHA remained more stable in CURDHA-ME, with only a 13.5% loss being observed after 3 months.

Accordingly, as shown in Table 2, the amount of isoprostanes (IsoP) and neuroprostanes (NeuroP) was much higher in DHA-ME than in CURDHA-ME after 3 months of storage at 4 °C under nitrogen.

**Figure 1:**
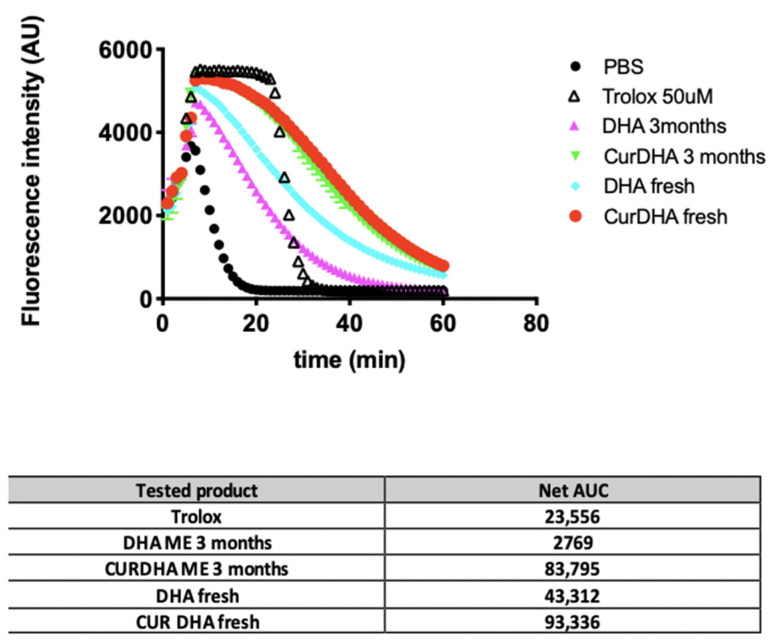
Comparison of the antioxidant potential of DHA- and CURDHA-MEs using the ORAC assay. Fluorescence decay curves of DHA and CURDHA-MEs. In the Table, the net AUC for Trolox and MEs were calculated by subtracting the blank (PBS) AUC value.

We also compared the antioxidant potential of DHA-ME and CURDHA-ME using the ORAC assay. As shown in Figure 1, we observed that the antioxidant potential of DHA-ME is lower than that of CURDHA-ME. The antioxidant potential of CURDHA-ME remained stable after 3 months of storage at 4 °C while that of DHA-ME decreased under the same storage conditions.

### 3.2. Effect of MEs on Short-Term Spatial Working Memory in the Aβ_25–35_ Model

**Figure 2:**
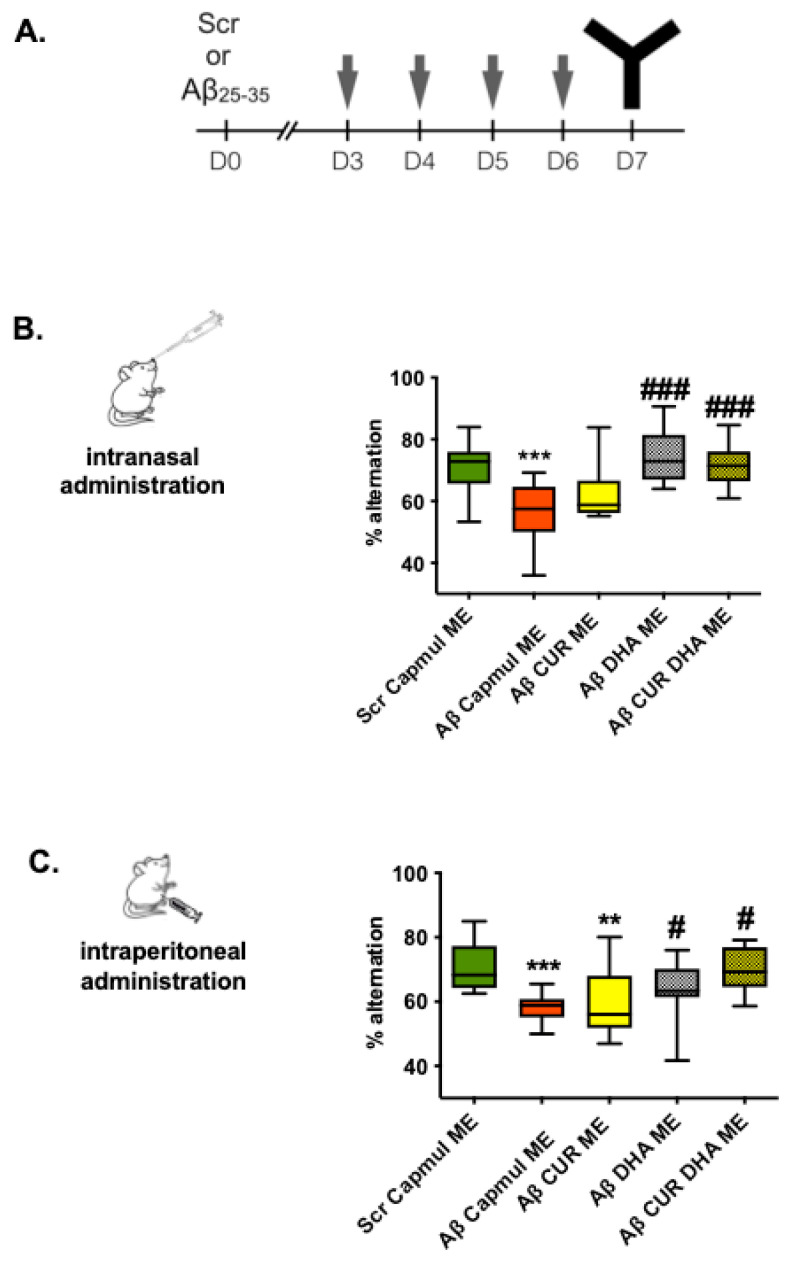
Effect of intranasal or intraperitoneal administration of CUR-, DHA-, and CURDHA-MEs on working memory in oAβ_25–35_ injected mice. (**A**) Timeline of ME administrations and Y-Maze task performed in oAβ_25–35_-injected (Aβ) and scrambled peptide-injected (Scr) mice. Spatial working memory was assessed in the Y-Maze 7 days after intracerebroventricular peptide injection (**B**) Effect of intranasal administration of MEs was measured the day after 4 days’ treatments (One-Way ANOVA followed by a Tukey post-hoc test, *** *p* < 0.001 vs. Scr mice, ### *p* < 0.001 vs. Aβ Capmul ME mice) (**C**) Effect of intraperitoneal administration of MEs was measured the day after 4 days’ treatments (One-Way ANOVA followed by a Tukey post-hoc test, *** *p* < 0.001, ** *p* < 0.01 vs. Scr mice, # *p* < 0.05 vs. Aβ Capmul ME mice). Scr and Aβ mice received a ME containing Capmul oil as vehicle. All the results express the percentage of alternation and are represented as whiskers (min to max).

We next evaluated the ability of intraperitoneal or intranasal administration of CUR-ME, DHA-ME, or CURDHA-ME to reverse oAβ_25–35_-induced cognitive deficits in mice. The animals were treated with ME during 4 consecutive days, starting 3 days after the icv injection of oAβ_25–35_ or ScAβ_25–35_ (Figure 2A). Supplementary Figure S1A,B illustrates the effect of Capmul-ME, CUR-ME, DHA-ME, and CURDHA-ME in ScAβ_25–35_-injected animals, with no effect observed on short-term spatial memory.

In contrast, the animals presented marked deficits seven days after oAβ_25–35_ injection (Aβ Capmul ME group; Figure 2B,C). Both intraperitoneal and intranasal administration of DHA-ME or CURDHA-ME were able to restore spatial memory deficits induced by oAβ_25–35_ injection to control values, while CUR-ME treatment was ineffective. As shown in Supplementary Figure S2A,B, no differences in total mobility in the Y-maze were measured between the groups.

Supplementary Figure S3 shows the impact of DHA-ME storage on its therapeutic effect. We observed a gradual decrease in DHA-ME efficiency over a few weeks of storage at 4 °C under nitrogen, while CURDHA-ME efficiency remained constant over the same period (data not shown).

### 3.3. Effect of MEs on Neuroinflammation, Oxidative Stress, and Tau Phosphorylation Markers in the Aβ_25–35_ Model

**Figure 3:**
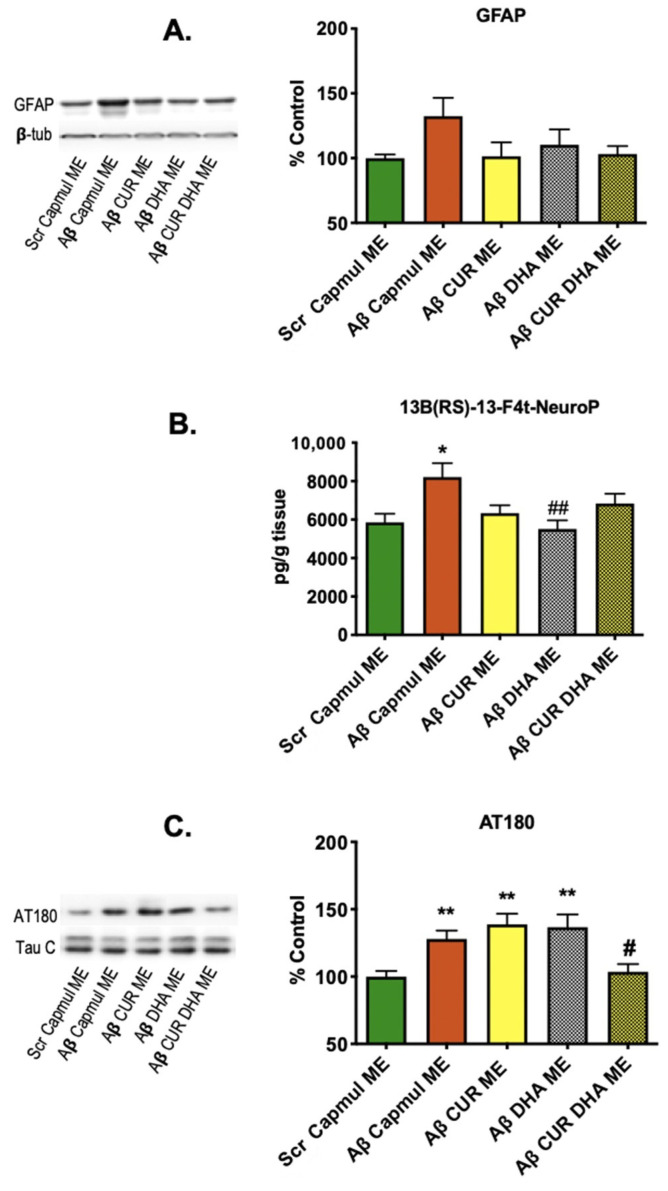
Effect of intranasal administration of CUR-, DHA-, and CURDHA-MEs on GFAP, neuroprostanes, and AT180 levels in oAβ_25–35_ injected mice. (**A**) Effect of intranasal administration of MEs on inflammation by immunoblot analysis of GFAP on hippocampal lysates. Results are normalized to β-tubulin levels. (**B**) Effect of intranasal administration of MEs on neuroprostane levels (13B(RS)-13-F4t-NeuroP) measured by LC-MS/MS on prefrontal cortices lysates (One-Way ANOVA followed by a Tukey post-hoc test, * *p* < 0.05 vs. Scr mice, ## *p* < 0.01 vs. Aβ Capmul ME mice). (**C**) Effect of intranasal administration of MEs on Tau hyperphosphorylation by immunoblot analysis of the phospho-epitope AT180 on hippocampal lysates (One-Way ANOVA followed by a Tukey post-hoc test, ** *p* < 0.01 vs. Scr mice, # *p* < 0.05 vs. Aβ Capmul ME mice). Results are normalized to Tau-C levels. Scr and Aβ mice received a ME containing Capmul oil as vehicle. All the results represent the percentage of the control group.

Next, we evaluated the impact of CUR-ME, DHA-ME, or CURDHA-ME intranasal administration on neuroinflammation, oxidative stress, and Tau phosphorylation markers in the brain tissue. As shown in Figure 3A,B, oAβ_25–35_ injection induced an increase of the astrocytic activation marker GFAP. All MEs tended to reduce GFAP amounts, although no significant difference was measured. Regarding oxidative stress, the amount of F_4_-NeuroP increased significantly (+40%) in oAβ_25–35_-injected mice. This increase was prevented by all ME formulations, although the effect was significant only with DHA-ME. We also investigated the impact of intranasal DHA on another hallmark of AD pathology, i.e., Tau phosphorylation, that can lead to the formation of paired helical filaments and neurofibrillary tangles in humans. A significant increase (+28%) in Tau phosphorylation (measured using AT180 antibodies) was measured in brain extracts from oAβ_25–35_-injected mice. Although CUR-ME and DHA-ME were ineffective to modify Tau phosphorylation levels, CURDHA-ME was able to fully restore this parameter in oAβ_25–35_-injected mice (Figure 3C).

### 3.4. Effect of CURDHA-MEs on Cognitive Impairment in the J20 Model

**Figure 4:**
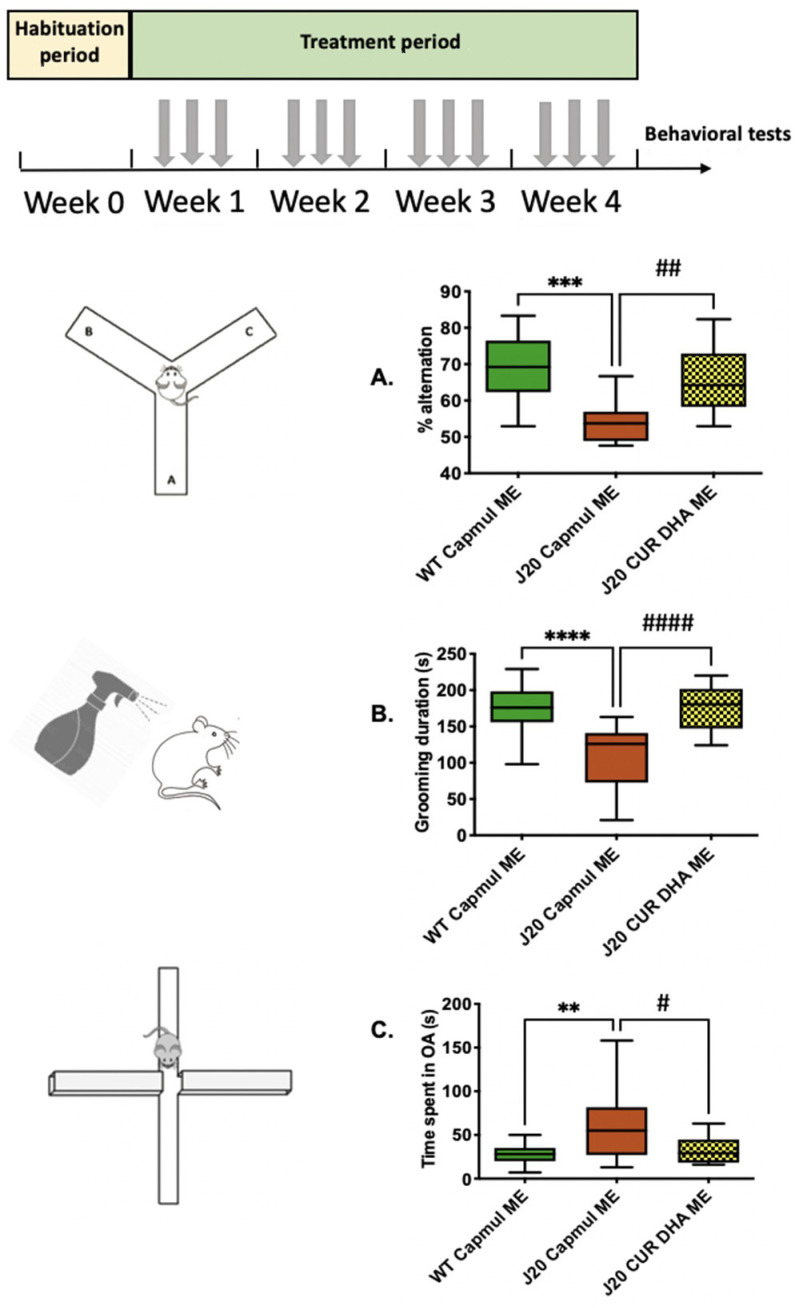
Effect of intranasal administration of CURDHA-ME on memory, depressive-like behavior, and anxiety in transgenic J20 mice. (**A**) Effect of intranasal administration of CURDHA-ME on working memory was measured in the Y-maze after a 4 week-treatment (One-Way ANOVA followed by a Tukey post-hoc test, *** *p* < 0.001 vs. WT mice, ## *p* < 0.01 vs. J20 Capmul ME mice). (**B**) Effect of intranasal administration of CURDHA-ME on depressive-like behavior was measured using the splash test after a 4 week-treatment (One-Way ANOVA followed by a Tukey post-hoc test, **** *p* < 0.0001 vs. WT mice, #### *p* < 0.0001 vs. J20 Capmul ME mice). (**C**) Effect of intranasal administration of CURDHA-ME on anxiety was measured in the O-Maze after a 4 week-treatment (One-Way ANOVA followed by a Tukey post-hoc test, ** *p* < 0.01 vs. WT mice, # *p* < 0.05 vs. J20 Capmul ME mice). WT and J20 mice received a micro-emulsion containing Capmul oil as vehicle. All the results are represented as whiskers (min to max).

In a further attempt to investigate the therapeutic potential of the intranasal administration of CURDHA-ME, we used a complementary, chronic AD model, i.e., hAPP^SwInd^-overexpressing J20 mice [30]. Three-month-old mice were treated over a four-week period as detailed under ‘Materials and Methods’. As shown in Figure 4, J20 mice displayed altered short-term memory, a depressive-like behaviour, and reduced anxiety at the age of three months. The intranasal administration of CURDHA-ME was able to restore all these parameters to control values after 4 weeks. The total mobility was not significantly different between WT and J20 mice (Supplementary Figure S2C).

### 3.5. Effect of CURDHA-MEs on Neuroinflammation, Oxidative Stress, and Tau Phosphorylation Markers in the J20 Model

**Figure 5:**
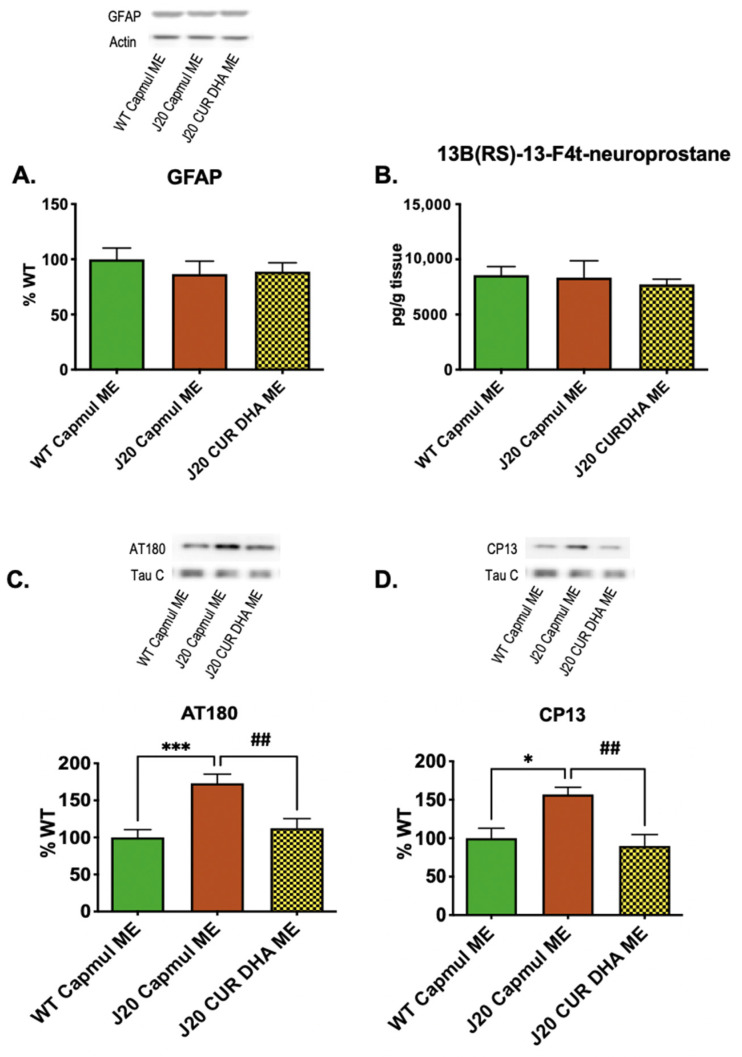
Effect of intranasal administration of CURDHA-ME on GFAP, neuroprostanes and Tau phosphorylation levels in transgenic J20 mice. (**A**) Effect of intranasal administration of CURDHA-ME on inflammation by immunoblot analysis of GFAP of hippocampal lysates from J20 mice. Results are normalized to β-tubulin levels. Results are represented as percentage of the control (WT Capmul ME). (**B**) Effect of intranasal administration of CURDHA-ME on neuroprostane levels (13B(RS)-13-F4t-NeuroP by LC-MS/MS) in prefrontal cortices lysates from J20 mice. WT and J20 mice received a ME containing Capmul oil as vehicle. Results are represented as mean ± SEM. (**C**,**D**) Effect of intranasal administration of CURDHA-ME on Tau phospho-epitopes AT180_(Thr231)_ (**C**) and CP13_(Ser202)_ (**D**) by immunoblot analysis of hippocampal lysates from J20 mice. (One-Way ANOVA followed by a Tukey post-hoc test, *** *p* < 0.0001, * *p* < 0.05 vs. WT mice, ## *p* < 0.01 vs. J20 Capmul ME mice). WT and J20 mice received a ME containing Capmul oil as vehicle. All the results are normalized to total Tau level (Tau-C) and represent the percentage of the control (WT Capmul ME).

Biochemical assays performed on brain extracts revealed that neither the neuroinflammatory marker GFAP, nor the oxidative stress marker 13 (*RS*)-F_4t_-NeuroP were increased in four months-old J20 mice, and CURDHA-ME administration had no impact on these markers (Figure 5A,B).

**Figure 6:**
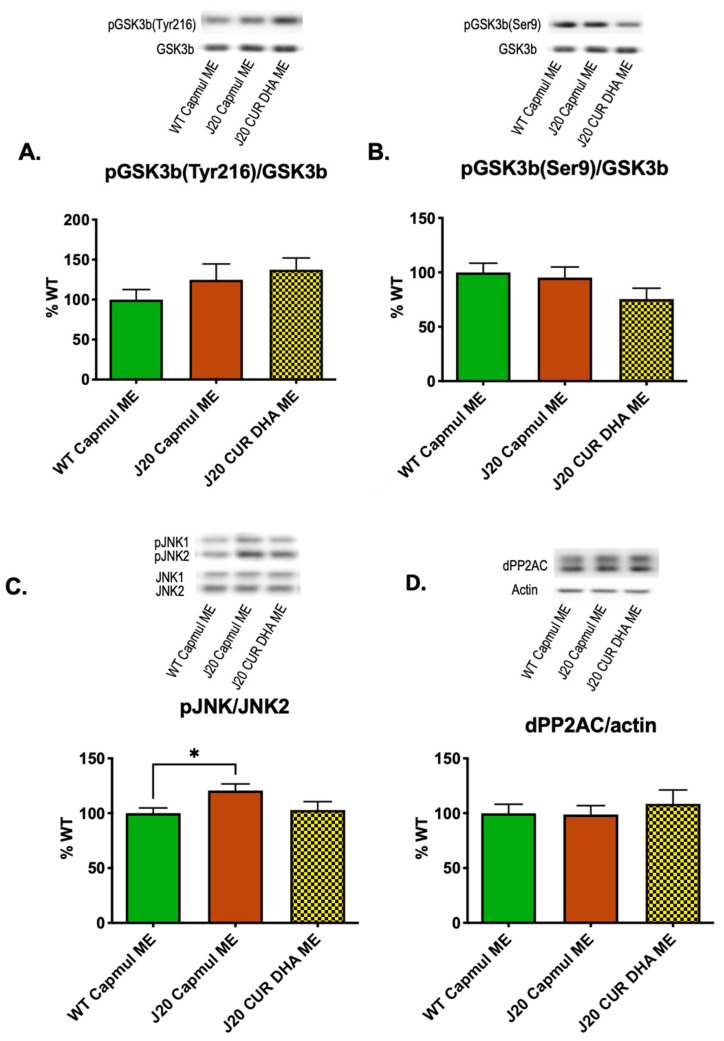
Effect of intranasal administration of CURDHA-ME on Tau kinases and phosphatase in transgenic J20 mice. Effect of intranasal administration of CURDHA-ME on GSK3β phospho-epitope Tyr216 levels that reflect its activation (**A**) and Ser9 levels that reflect its inactivation (**B**) by immunoblot analysis of hippocampal lysates from J20 mice. (**C**) Effect of intranasal administration of CURDHA-ME on Phospho-JNK levels by immunoblot analysis of hippocampal lysates from J20 mice (One-Way ANOVA followed by a Tukey post-hoc test, * *p* < 0.05 vs. WT mice). (**D**) Effect of intranasal administration of CURDHA-ME on the major Tau phosphatase, PP2AC, by immunoblot analysis of hippocampal lysates from J20 mice. The demethylated/total PP2AC ratio was calculated as a reflection of its inactivation. WT and J20 mice received a ME-containing Capmul oil as a vehicle. All the results are normalized to total corresponding protein levels and expressed as the percentage of the control (WT CAPMUL ME).

In contrast to neuroinflammation and oxidative stress, Tau phosphorylation as measured using AT180 and CP13 antibodies was significantly increased in four-month-old J20 mice, as shown in Figure 5C,D. CURDHA-ME treatment allowed the restoration of Tau phosphorylation to control values on both residues. In this model we further thought to determine whether increased Tau phosphorylation may result from an increased activation of the GSK3-β kinase. As shown in Figure 6A,B, we observed no alteration of GSK3-β phosphorylation on Tyr216 and Ser9 residues, suggesting its activity is not modified in four-month-old J20 mice. Another major kinase involved in Tau phosphorylation is JNK. A significant increase in JNK2 phosphorylation was measured in brain hippocampi from four-month-old J20 mice, and this increase was reversed in CURDHA-ME-treated animals (Figure 6C). We then analyzed the methylation status of the catalytic C subunit of the protein phosphatase 2 A (PP2A), the main phosphatase involved in Tau dephosphorylation; no alteration of PP2AC methylation status was measured in three-month-old J20 mice, suggesting an unaltered PP2AC activity (Figure 6D).

## 4. Discussion

There is, at this time, a lack of consensus on the benefits of DHA supplementation for cognition in AD patients [37,38]. This probably stems from the fact that after oral administration, DHA has to undergo a long metabolic pathway before reaching the central nervous system, meaning there can be important inter-individual variations in brain supply [39]. The present study was designed to explore, for the first time, a route of administration for DHA that bypasses the gastro-intestinal tract and the BBB, i.e., the intranasal administration route. We demonstrated using two complementary mouse AD models—a non-transgenic acute model and a transgenic chronic model—that the intranasal administration of a DHA-enriched microemulsion efficiently prevents cognitive decline and blocks Tau hyperphosphorylation through its action on JNK signalling. The acute model was used to screen MEs and choose the most efficient form, while the chronic model allowed us to evaluate the impact of our treatment strategy on endogenous Aβ-induced toxicity.

In the first part of this study, we measured the stability of DHA in MEs containing Curcumin or not containing it during storage at 4 °C. A rapid loss of DHA was observed in DHA-MEs, while a constant level remained in CURDHA-MEs (Table 1). Accordingly, the non-enzymatic oxidation of DHA to isoprostanes and F_4_-neuroprostanes was much lower in CURDHA-MEs than in DHA-MEs (Table 2). MEs containing both DHA and Curcumin had the highest antioxidant capacity in vitro, and no decrease was measured during storage at 4 °C (Figure 1). Taken together, these observations indicate that DHA is not protected from oxidative modifications when not combined to another antioxidant in MEs. This finding is in accordance with earlier studies, showing that DHA oxidation can occur even in microemulsions [40]. In accordance with our results, a recent study by Shehzad et al. showed that the co-encapsulation of Curcumin and Resveratrol, which act as co-antioxidants inside nanoemulsions, is efficient enough to reduce DHA oxidative modifications that may lead to the formation of toxic end-products like 4-hydroxyhexenal (4-HHE) [41].

We next compared the effects of the intraperitoneal and intranasal administration of CUR-ME, DHA-ME, and CURDHA-ME on oAβ-induced short-term spatial memory impairment. Both intraperitoneal and intranasal treatment with DHA-ME and CURDHA-ME were able to restore spatial memory to levels measured in control mice (Figure 2), while oral administration of either ME was ineffective (data not shown). Oral administration was used as a control to make sure that the observed effect using intranasal administration was not attributable to passage into the gastrointestinal tract. Intranasal drug administration is emerging as a promising strategy for the treatment of AD and our observations suggest for the first time it is suitable to deliver nanovectorized DHA to the brain. It is of note that the dose of DHA used for the treatment in the present study was around 10 mg/kg, which is much lower than the doses that are usually tested for dietary supplementation. The use of a lower dosage is important since it has been reported that high doses of antioxidants can lead to pro-oxidant effects [42]. Several studies have suggested that dietary DHA supplementation can counteract AD pathology in animal models, and cohort studies have revealed that the consumption of DHA is associated with a reduced risk of cognitive decline in middle-aged or aged populations [43,44]. However, supplementation studies conducted in humans did not allow us to conclude about the therapeutic potential of DHA for the prevention or treatment of AD, and our hypothesis is that this inconsistency can been explained by the long metabolic pathway DHA has to undergo before reaching the brain when administered through diet [45]. In particular, the BBB transport of DHA is governed by several transporters (fatty acid binding protein FABP5 [46], fatty acid transport proteins (FATP-1 and FATP-4) [47] and Mfsd2a [48]), whose activity seems to be modulated in animal AD models. Thus, Pan et al. demonstrated that the BBB transport of DHA is reduced in APP/PS1 mice, due to reduced FABP5 expression at the BBB. The deficiency at the BBB of FABP5, and perhaps FATP-1 and FATP-4, is thought to affect APP/PS1 mice. Indeed, they are more vulnerable to DHA deficit, with reduced DHA access into the CNS and impairments in short-term spatial memory and recognition memory. Although there are no available data on the expression of these receptors in AD patients, it is noteworthy that brain uptake of DHA was reported to be inefficient in carriers of ApoE4, who are prone to the earlier onset of AD [49]. Thus, we believe that using the intranasal route of administration, which allows the bypass of the BBB and provides direct access to the brain, could make the difference to firmly demonstrate the potential of DHA to prevent neurodegeneration.

To address the question of the impact of DHA oxidation on ME efficiency, we compared three preparations that had been opened and conserved at 4 °C under nitrogen for 1, 2, or 3 months. Although there is evidence that the metabolites formed through enzymatic or non-enzymatic DHA oxidation may play a role in modulating oxidative homeostasis in cells [50,51], our data showed a gradual loss of efficiency between fresh MEs and MEs that had been stored for several weeks (Supplementary Figure S3). This finding has two implications: first, DHA oxidation needs to be prevented to ensure protection against cognitive loss under our experimental setting; and second, DHA oxidation during the digestion process may well constitute another issue, thereby leading to the unpredictable effects of DHA supplementation and frequent discrepancies between clinical studies. In the same order of this idea, Grimm et al. showed that only 1% DHA oxidation is able to revert the protective effect of DHA and to increase Aβ production [52].

Next, we evaluated the effects of CURDHA-ME treatment on cognitive loss in the J20 AD animal model. This mouse model is characterized by an early impairment of spatial memory, a depressive-like behaviour, and reduced anxiety [53,54]. After a 4-week-treatment (3 days/week), the Y-maze, splash, and O-maze tests were performed on 4-month-old J20 mice. In this transgenic AD model, CURDHA-ME treatment remarkably increased the performances in all paradigms, indicating improved spatial working memory, as well as normalized anxiety and depressive states (Figure 4).

We explored the impact of ME treatment on several parameters associated with Aβ toxicity. Both oxidative stress and Tau phosphorylation were significantly increased in oAβ_25–35_-injected mice, while a non-significant increase was observed for the neuroinflammation marker GFAP. All MEs restored neuroinflammatory and oxidative stress markers to normal values (Figure 3A,B), which is in accordance with the well-documented antioxidant and anti-inflammatory properties of CUR and DHA. It is interesting to note that CUR-MEs were as effective as DHA-MEs and CURDHA-MEs at reducing GFAP expression and oxidative stress, while having no beneficial impact on memory. In four-month-old J20 mice, no increase of neuroinflammation and oxidative stress was detectable yet, thus the impact of MEs on these parameters could not be evaluated (Figure 5A,B).

Beyond oxidative stress and neuroinflammation, it is now well recognized that Tau pathology in the brain is closely related to clinical manifestations. Tau hyperphosphorylation can lead to its aggregation and neurofibrillary tangle formation [55]. In a meta-analysis of cognitive decline in preclinical 3×Tg AD models, Tauopathy was found to be much more impactful than Aβ, with strong and significant correlations being measured between several phosphorylated Tau isoforms and cognitive performance in both the Morris water maze and novel object recognition tests [56]. The same is true in humans, as recently reported in 1431 older adults spanning from cognitively normal to AD dementia, whose tangle load predicted falling mini-mental state exam scores better than plaque load or cortical thickness did [57]. It has been recently proposed that Tau pathology, which arises about a decade before the appearance of Aβ deposits in human brains, could be a key initiating event in sporadic AD [58]. Both DHA and CUR were previously shown to be potent inhibitors of Tau phosphorylation [59]. The impact of CUR-, DHA-, and CURDHA-MEs on Tau phosphorylation at pThr231 was addressed in the present study in an attempt to explain their differential effects on oAβ_25–35_-induced short-term spatial memory impairment. The phosphorylation of pThr231 is an early event in AD [60], being associated with neurofibrillary tangle pathology in Braak stages [61,62], and its quantification in the CSF can be used for diagnosis purposes [63]. Interestingly, although both DHA-ME and CURDHA-ME were able to improve spatial memory, only CURDHA-ME had a significant impact on Tau phosphorylation at the pT231 phospho-epitope (AT180) (Figure 3C), which was confirmed in the transgenic AD model (Figure 5C). Moreover, the treatment also reduced Tau phosphorylation at pSer202 (CP13), an early marker of AD and major contributor to NFT formation [64,65] (Figure 5D). Thus, the reduction of Tau phosphorylation at these epitopes with CURDHA-ME could be a valid therapeutic strategy for the prevention of AD and other Tauopathies.

Tau phosphorylation is tightly regulated through the action of several kinases and phosphatases. GSK3β is a key Tau kinase, and CUR has been previously shown to be a strong inhibitor of its activity. We thus hypothesized that reduced Tau phosphorylation could have resulted from the treatment-induced inhibition of GSK3β. However, we observed no alteration of GSK3β phosphorylation at Ser9 (inactive form), nor at Tyr216 (active form) residues in our four-month-old J20 mice, suggesting that altered GSK3β activity is not responsible for the increased Tau phosphorylation in this context (Figure 6A,B). Beyond GSK3β, c-Jun N-terminal kinases (JNKs) have been shown to play a critical role in driving the hyperphosphorylation of Tau in cell culture systems, AD models, and in the brain of AD patients [66,67,68,69]. JNK is currently gaining attention as a new therapeutic target. Both Thr231 (AT180) and Ser202 (CP13) Tau phosphorylation sites are impacted by JNK activity [70,71]. JNK activation is induced by reactive oxygen species [72] and observed in neurons and dystrophic neurites of the AD model mice and AD brain in which it progressively overlaps Tau-positive neurofibrillary pathology [73,74]. In contrast, JNK inhibition was shown to be associated with cognitive improvement and neuroprotective effects in 5XFAD mice [75]. The inhibition of JNK stands as an important mechanism responsible for the beneficial effects of ω-3 PUFAs. In a recent study by Vela et al., DHA was shown to inhibit cognitive deficits in a mouse model of AD through this mechanism [76] and several polyphenols, including CUR, were also shown to be potent JNK inhibitors [77,78]. In the present study, a significant increase of JNK2 phosphorylation was observed in J20 mice, which was abolished in CURDHA-ME-treated animals (Figure 6C). Thus, the intranasal administration of CURDHA-MEs efficiently inhibited Tau phosphorylation, and our results suggest this may be related to the inhibition of JNK phosphorylation and signalling.

## 5. Conclusions

In conclusion, we demonstrated for the first time that intranasal administration of nanovectorized DHA is a powerful strategy to counteract Tau hyperphosphorylation and cognitive loss in murine AD models. These results pave the way for the development of a new approach to target the brain with DHA for the prevention or treatment of this devastating disease. Our results also emphasized the necessity to prevent DHA oxidation to maintain efficacy through the addition of a co-antioxidant as CUR.

## Figures and Tables

**Table 1:** DHA levels (mg/mL) in DHA and CUR-DHA loaded microemulsions during storage at 4 °C.

Months After Preparation	DHA ME	CURDHA ME
0	20.00	21.81
1	18.23	21.49
2	10.90	20.13
3	4.10	18.87

**Table 2:** Oxylipin levels (pg/2 mg DHA) in DHA and CUR-DHA loaded microemulsions stored 3 months at 4 °C under nitrogen.

	DHA ME	CURDHA ME
15-A2t-IsoP	1367 ± 82	304 ± 20 **
5(S)-5-F3t-IsoP	1504 ± 134	1015 ± 189 **
4(RS)-4-F4t-NeuroP	11,276 ± 1464	6986 ± 755 **
13B(RS)-13-F4t-NeuroP	4282 ± 252	2658 ± 295 **

** *p* < 0.05.

## Data Availability

All of the data is contained within the article and supplementary materials.

## Supplementary Materials

The following are available online at https://www.mdpi.com/article/10.3390/antiox11050838/s1, Figure S1: Effect of intranasal or intraperitoneal administration of CUR, DHA and CURDHA MEs on working memory in scrambled Aβ_25–35_ peptide-injected mice. Figure S2: Effect of intranasal or intraperitoneal administration of CUR, DHA and CURDHA-MEs on locomotion in oAβ_25–35-_injected mice and J20 mice. Figure S3: Effect of intranasal administration of DHA-MEs after different times of storage on working memory in oAβ_25–35_-injected mice.

## Abbreviations

AD: Alzheimer’s disease; CUR, curcumin; DHA, docosahexaenoic acid; GFAP, glial fibrillary acid protein; IsoP, isoprostanes; JNK, c-Jun N-terminal kinase; ME, microemulsion; NeuroP, neuroprostanes; oAβ_25–35_, oligomeric amyloid beta 25–35 peptide; PUFA, polyunsaturated fatty acid; NEO-PUFAs, non-enzymatic oxidation product of polyunsaturated fatty acids; PP2A, protein phosphatase 2A; Scr, scrambled; WT, wild type.
