# Supplementary material for: Intranasal Administration of Nanovectorized Docosahexaenoic Acid (DHA) Improves Cognitive Function in Two Complementary Mouse Models of Alzheimer’s Disease
Source: Antioxidants (Basel). 2022 Apr 25;11(5):838. doi: 10.3390/antiox11050838 (PMC9137520; doi:10.3390/antiox11050838)
Supplement: Supplementary file 1 [file antioxidants-11-00838-s001.zip › antioxidants-1640807-supplementary.pdf]

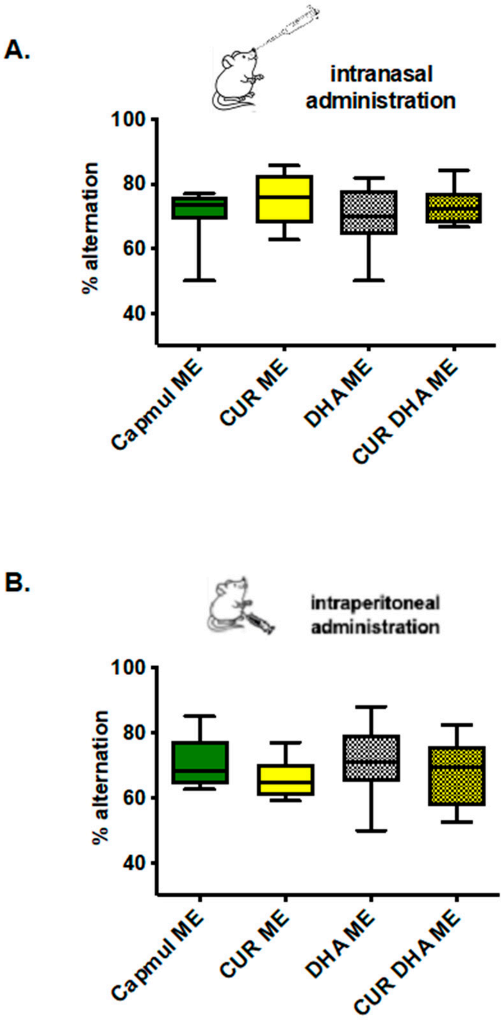

Suppl. Figure S1: Effect of intranasal or intraperitoneal administration of CUR, DHA and CURDHA MEs on working memory in scrambled A$\beta_{25\text{-}35}$ peptide-injected mice. Effect of intranasal (**A**) or intraperitoneal (**B**) administration of MEs on spatial working memory was measured after 4 days of treatment with either Capmul ME (vehicle), CUR-ME, DHA-ME or CURDHA-ME in scrambled A$\beta_{25\text{-}35}$ peptide-injected mice. All the results represent the percentage of alternation and are expressed as whiskers (min to max). No significant differences were observed (One-Way ANOVA).

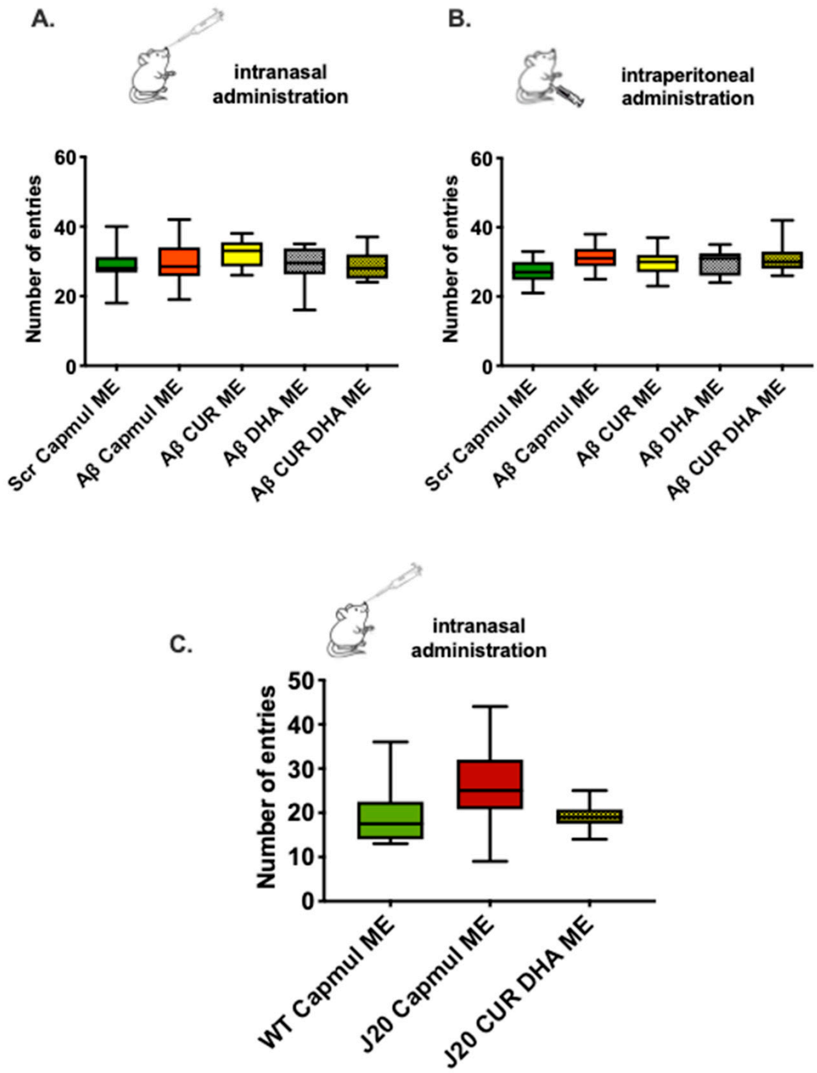

Suppl. Figure S2: Effect of intranasal or intraperitoneal administration of CUR, DHA and CURDHA-MEs on locomotion in $oA\beta_{25-35}$-injected mice and J20 mice. Locomotion (number of entries in the Y-Maze) was assessed 7 days after intracerebroventricular peptide injection in $oA\beta_{25-35}$ injected mice or in 4-months old J20 mice. A & B. Effect of intranasal (**A**) or intraperitoneal (**B**) administration of MEs was measured the day after 4 days' treatments in $oA\beta_{25-35}$ injected mice. C. Effect of intranasal administration of MEs was measured after a 4 week-treatment in J20 mice. Capmul ME was used as a vehicle. All the results represent the number of entries and are expressed as whiskers (min to max). No significant differences were observed (One-Way ANOVA).

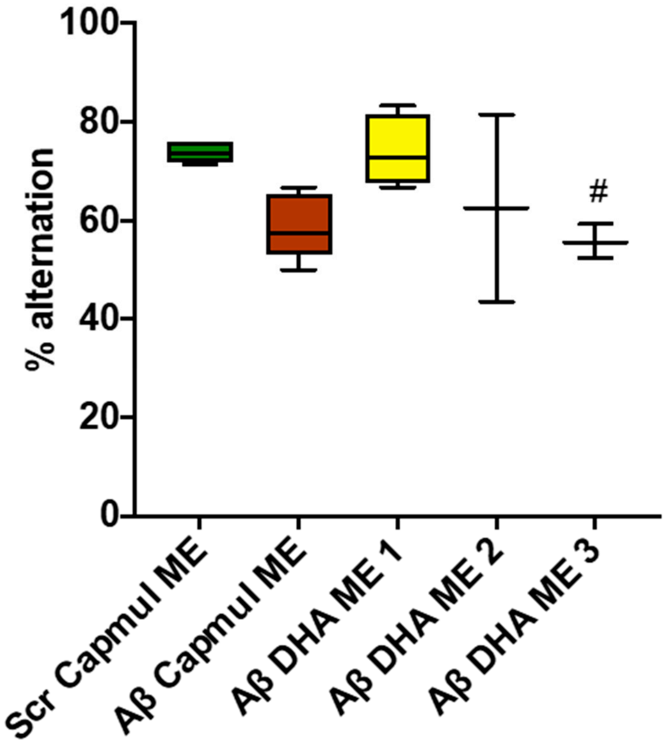

Suppl. Figure S3: Effect of intranasal administration of DHA-MEs after different times of storage on working memory in oAβ_{25-35}-injected mice. Spatial working memory was assessed in the Y-Maze 7 days after intracerebroventricular scrambled (Scr) or oAβ_{25-35} (Aβ) peptide injection. Effect of intranasal administration of DHA MEs after different times (1, 2 or 3 months) of storage at 4°C was measured after 4 days of treatment (One-Way ANOVA followed by a Tukey post-hoc test, $^{\#}p<0.05$ *vs* Scr Capmul ME group). Scrambled and Aβ-injected mice received a ME containing Capmul oil as vehicle. All the results represent the percentage of alternation and are expressed as whiskers (min to max).
